# Determinants of HIV late presentation among men who have sex with men in Portugal (2014–2019): who’s being left behind?

**DOI:** 10.3389/fpubh.2024.1336845

**Published:** 2024-02-29

**Authors:** Ricardo Abrantes, Victor Pimentel, Mafalda N. S. Miranda, Ana Rita Silva, António Diniz, Bianca Ascenção, Carmela Piñeiro, Carmo Koch, Catarina Rodrigues, Cátia Caldas, Célia Morais, Domitília Faria, Elisabete Gomes da Silva, Eugénio Teófilo, Fátima Monteiro, Fausto Roxo, Fernando Maltez, Fernando Rodrigues, Guilhermina Gaião, Helena Ramos, Inês Costa, Isabel Germano, Joana Simões, Joaquim Oliveira, José Ferreira, José Poças, José Saraiva da Cunha, Jorge Soares, Sandra Fernandes, Kamal Mansinho, Liliana Pedro, Maria João Aleixo, Maria João Gonçalves, Maria José Manata, Margarida Mouro, Margarida Serrado, Micaela Caixeiro, Nuno Marques, Olga Costa, Patrícia Pacheco, Paula Proença, Paulo Rodrigues, Raquel Pinho, Raquel Tavares, Ricardo Correia de Abreu, Rita Côrte-Real, Rosário Serrão, Rui Sarmento e Castro, Sofia Nunes, Telo Faria, Teresa Baptista, Daniel Simões, Luis Mendão, M. Rosário O. Martins, Perpétua Gomes, Marta Pingarilho, Ana B. Abecasis, the BESTHOPE Study Group

**Affiliations:** ^1^Global Health and Tropical Medicine (GHTM), Associate Laboratory in Translation and Innovation Towards Global Health (LA-REAL), Institute of Hygiene and Tropical Medicine, NOVA University of Lisbon (IHMT/UNL), Lisbon, Portugal; ^2^Serviço de Infeciologia, Hospital Beatriz Ângelo, Loures, Portugal; ^3^U. Imunodeficiência, Hospital Pulido Valente, Centro Hospitalar Universitário de Lisboa Norte, Lisbon, Portugal; ^4^Serviço de Infeciologia, Centro Hospitalar de Setúbal, Setúbal, Portugal; ^5^Serviço de Doenças Infeciosas, Centro Hospitalar Universitário de São João, Porto, Portugal; ^6^Centro de Biologia Molecular, Serviço de Imunohemoterapia do Centro Hospitalar Universitário de São João, Porto, Portugal; ^7^Serviço de Medicina 1.4, Hospital de São José, Centro Hospitalar Universitário de Lisboa Central, Lisbon, Portugal; ^8^Serviço de Patologia Clínica, Centro Hospitalar e Universitário de Coimbra, Coimbra, Portugal; ^9^Serviço de Medicina 3, Hospital de Portimão, Centro Hospitalar Universitário do Algarve, Portimão, Portugal; ^10^Unidade Local de Saúde do Baixo Alentejo, Hospital José Joaquim Fernandes, Beja, Portugal; ^11^Serviço de Medicina 2.3, Hospital de Santo António dos Capuchos, Centro Hospitalar de Lisboa Central, Lisbon, Portugal; ^12^Hospital de Dia de Doenças Infeciosas, Hospital Distrital de Santarém, Santarém, Portugal; ^13^Serviço de Doenças Infeciosas, Hospital Curry Cabral, Centro Hospitalar de Lisboa, Lisbon, Portugal; ^14^Serviço de Patologia Clínica, Hospital de Sta Maria, Centro Hospitalar Universitário de Lisboa Norte, Lisbon, Portugal; ^15^Serviço de Patologia Clínica, Centro Hospitalar do Porto, Porto, Portugal; ^16^Laboratório de Biologia Molecular (LMCBM, SPC, CHLO-HEM), Lisbon, Portugal; ^17^Serviço de Infeciologia, Centro Hospitalar e Universitário de Coimbra, Coimbra, Portugal; ^18^Serviço de Medicina 2, Hospital de Faro, Centro Hospitalar Universitário do Algarve, Faro, Portugal; ^19^Serviço de Doenças Infeciosas, Hospital de Egas Moniz, Centro Hospitalar de Lisboa Ocidental, Lisbon, Portugal; ^20^Serviço de Infeciologia, Hospital Garcia da Orta, Almada, Portugal; ^21^Serviço de Infeciologia, Centro Hospitalar do Porto, Porto, Portugal; ^22^Serviço de Infeciologia, Hospital de Aveiro, Centro Hospitalar Baixo Vouga, Aveiro, Portugal; ^23^Serviço de Infeciologia, Hospital Dr. Fernando da Fonseca, Amadora, Portugal; ^24^Serviço de Patologia Clínica, Biologia Molecular, Centro Hospitalar Universitário de Lisboa Central, Lisbon, Portugal; ^25^Serviço de Infeciologia, Hospital de Faro, Centro Hospitalar Universitário do Algarve, Faro, Portugal; ^26^Serviço de Infeciologia, Unidade de Local de Saúde de Matosinhos, Hospital Pedro Hispano, Matosinhos, Portugal; ^27^Grupo de Ativistas em Tratamentos (GAT), Lisbon, Portugal; ^28^Egas Moniz Center for Interdisciplinary Research (CiiEM), Egas Moniz School of Health & Science, Almada, Portugal

**Keywords:** HIV-1, men who have sex with men, late presentation, drug resistance, Portugal, vulnerable populations

## Abstract

**Introduction:**

HIV late presentation (LP) remains excessive in Europe. We aimed to analyze the factors associated with late presentation in the MSM population newly diagnosed with HIV in Portugal between 2014 and 2019.

**Methods:**

We included 391 newly HIV-1 diagnosed Men who have Sex with Men (MSM), from the BESTHOPE project, in 17 countrywide Portuguese hospitals. The data included clinical and socio-behavioral questionnaires and the viral genomic sequence obtained in the drug resistance test before starting antiretrovirals (ARVs). HIV-1 subtypes and epidemiological surveillance mutations were determined using different bioinformatics tools. Logistic regression was used to estimate the association between predictor variables and late presentation (LP).

**Results:**

The median age was 31 years, 51% had a current income between 501–1,000 euros, 28% were migrants. 21% had never been tested for HIV before diagnosis, with 42.3% of MSM presenting LP. 60% were infected with subtype B strains. In the multivariate regression, increased age at diagnosis, higher income, lower frequency of screening, STI ever diagnosed and higher viral load were associated with LP.

**Conclusion:**

Our study suggests that specific subgroups of the MSM population, such older MSM, with higher income and lower HIV testing frequency, are not being targeted by community and clinical screening services. Overall, targeted public health measures should be strengthened toward these subgroups, through strengthened primary care testing, expanded access to PrEP, information and promotion of HIV self-testing and more inclusive and accessible health services.

## 1 Introduction

HIV infection continues to significantly impact the health of millions of people in the WHO European Region. In the past 30 years, over this region, more than 2.2 million people have been diagnosed with HIV, and in 2021 106,508 people were newly diagnosed with HIV, with an incidence rate of 12 per 100,000 inhabitants (1).

In Portugal, between 1983 and 2022, 66,061 cases of HIV infection were diagnosed, of which 23,637 (35.8%) reached the AIDS stage (2). Although between 2013 and 2023, there was a 56% reduction in new HIV infection cases and a 74% in new AIDS cases, Portugal stands out for the high rates of new cases of HIV infection and AIDS among Western European countries (2). According to the latest surveillance report, in 2022, 804 new cases of HIV infection were diagnosed in Portugal, with an incidence rate of 7.7 cases per 100,000 inhabitants (2).

Men who have sex with men (MSM) are a priority group for the prevention and control of HIV infection. Sex between men remains the predominant mode of HIV transmission reported in the EU/EEA, accounting for 39% (5815) of all new HIV diagnoses in 2020 and more than half (53%) of diagnoses with known route of transmission (3). In Portugal, according to the report HIV and AIDS Infection – 2023, in 2022, 61.8% of HIV diagnoses were in MSM (2). Stigma and discrimination related to sexual orientation can indeed act as significant barriers to HIV testing and early care seeking among MSM (4, 5). Structural stigma and sexual orientation concealment reduces MSM’s access to HIV-preventive services, health literacy and prevention measures (6, 7).

Despite all global efforts to increase HIV testing, the percentage of late presentation (LP) remains consistently high across European countries (8), indicating the inadequacy of public health efforts to decrease LP and its impact on morbidity, mortality and risk of transmission (9). This is significant because even with antiretroviral therapy, LP (CD4 < 350 cells/μL or presence of AIDS-defining disease) (10) is the most important predictor of mortality with AIDS (11, 12). The present recommendations for MSM suggest undergoing testing at least once a year (13). Clearly insufficient, in Portugal, the most recent data indicates that only 65% of MSM without known infection have tested for HIV during the past 12 months (14). Thus, unsurprisingly, a study in a Portuguese hospital showed that 24.9% of MSM had HIV LP (15).

Given the above scenario, in this study, we aimed to analyze the factors associated with LP in the MSM population diagnosed with HIV in Portugal between 2014 and 2019.

## 2 Materials and methods

### 2.1 Ethics

This study was approved by the Ethics Committees of all participating hospitals.

### 2.2 Study population

Data was collected within the scope of the BESTHOPE project from MSM newly diagnosed with HIV-1 infection, who presented for care in 17 Portuguese hospitals countrywide between September 2014 and December 2019, had an antiretroviral drug resistance test before starting ART and were older than 18 years.

391 MSM were included in this study upon invitation to participate in the study by clinicians in the first appointment. This sample represents 19% of the total number of MSM diagnosed with HIV infection in Portugal in this period (16–21).

### 2.3 BEST HOPE project

The BESTHOPE project was an observational cross-sectional study using different data collection instruments: socio-behavioral questionnaires, clinical questionnaires and genomic sequences of the HIV-1 virus infecting patients followed in Portuguese hospitals countrywide, to understand the dynamics and the behavioral determinants of HIV transmission.

### 2.4 Data collection

Sociodemographic and behavioral data were collected with a survey questionnaire in the infectious diseases/internal medicine consultations, constructed by researchers in collaboration with patients and NGO members, and then completed by the participants. Clinicians provided clinical data. Viral genomic sequences from the first resistance test (before the start of ART) were collected from the patients’ records and included protease and reverse transcriptase sequences. The resulting database was coded and anonymized.

### 2.5 Late presenters

A CD4 count <350 cells/μL or an AIDS-defining event regardless of CD4 count at presentation for care was defined as Late Presentation (LP), and a CD4 count ≤ 200 cells/μL or an AIDS-defining event was described as Late Presentation with Advanced Disease (LPAD) (10).

### 2.6 HIV-1 subtyping

HIV-1 subtypes were determined using the patients´ genomic sequences, through the use of three different algorithms (REGA V.3.0, Comet e Scuel) (22, 23). The consensus of the three tools was considered or, when there was no consensus, the assignment of the majority of the subtyping tools was considered.

### 2.7 Drug resistance analyses

Sequences were submitted to the HIV Drug Resistance Database (Stanford University)^1^ to assess TDR. TDR was defined as the existence of one or more drug resistance mutations on surveillance (SDRMs), in accordance with the WHO 2009 surveillance list (24), which includes Protease Inhibitor (PI), Nucleoside Reverse Transcriptase Inhibitor (NRTI) and Non-Nucleoside Reverse Transcriptase Inhibitor (NNRTI) mutations.

### 2.8 Recentness of infection

The rate of ambiguity in viral genomic sequences has been suggested as a method to differentiate chronic vs. recent HIV-1 infection (25), which is useful to compare to other criteria namely the consensus definition (10) used in this study. Regarding the cutoff used, chronic infection was defined as an ambiguity rate > 0.45% and recent infection = < 0.45% (25).

### 2.9 Statistical analysis

For the descriptive analysis of sociodemographic and behavioral characteristics of MSM with HIV-1, MSM with LP, the median and the proportion of continuous variables and qualitative variables were calculated, respectively. For proportions, the 95% confidence interval was calculated and, for the medians, the interquartile range. To compare characteristics between MSM with and without LP, Student’s t-test, Mann–Whitney’s U-test, Chi-square test and Fisher’s exact test were used. Two separate logistic regression models were calculated: one to study the sociodemographic, behavioral, testing, prophylaxis and STIs factors associated with LP, and another to study clinical and viral genomics factors associated to LP. Factors in the univariate models with a *p-*value >0.2 were included in the multivariate model. We assessed the presence of multicollinearity by calculating modified generalized variance-inflation factors [GVIF (1/(2 × Df)); Fox and Monette] with a threshold of 2, and the goodness of fit for logistic regression models with the Hosmer–Lemeshow test. The significance level was 5%. Data analysis was performed in R(v4.2.2) (26).

## 3 Results

From the total of 391 MSM included in our sample with socio-demographic and behavioral data, 371 had clinical information that allowed the classification of presentation to care status. 58% (95% CI: 52–63%) were classified as NonLP and 42.3% (95% CI: 37–48%) were classified as LP.

### 3.1 Sociodemographics

In this population, the median age at diagnosis was 31 (IQR: 25–38) years, with MSM with LP (35, IQR: 26–43) being significantly older than Non LP (29, IQR: 24–35). Most men were Portuguese with a proportion of 72% (95% CI: 67–76%), followed by Latin American with 21% (95% CI: 17–25%). As for the district of residence, men lived mainly in Lisbon (53%), Porto (16%) and Faro (10%). Concerning the level of education and employment status, 42% (95% CI: 37–47%) secondary level (12th degree/technical specialization), and 43% (95% CI: 37–48%) higher education (bachelor, master, PhD), with 76% (95% CI: 71–80%) employed, with the majority having an income in the minimum wage range (501–1,000€) (51, 95% CI: 46–57%) (Table 1).

**Table 1:** Sociodemographic characteristics of the MSM overall, non-late presenters (NonLP) and late presenters (LP).

	*N*	Overall	NonLP (*N* = 214)	LP (*N* = 157)	*p*-value
		*N* (%) [95% CI] Median (IQR)	*N* (%) [95% CI] Median (IQR)	*N* (%) [95% CI] Median (IQR)	
Sociodemographics
Diagnosis year	370				0.3
2014–2016		246 (66%) [61,71%]	147 (69%) [62, 75%]	99 (63%) [55,71%]	
2017–2019		124 (34%) [29,39%]	67 (31%) [25,38%]	57 (37%) [29,45%]	
Age at diagnosis	370	31 (25, 38)	29 (24, 35)	35 (26, 43)	<0.001
Age at diagnosis (groups)	370				<0.001
18–24		78 (21%) [17, 26%]	54 (25%) [20, 32%]	24 (15%) [10, 22%]	
25–34		154 (42%) [37, 47%]	101 (47%) [40, 54%]	53 (34%) [27, 42%]	
35–44		90 (24%) [20, 29%]	41 (19%) [14, 25%]	49 (31%) [24, 39%]	
45–54		35 (9.5%) [6.8, 13%]	11 (5.1%) [2.7, 9.3%]	24 (15%) [10, 22%]	
≥55		13 (3.5%) [2.0, 6.1%]	7 (3.3%) [1.4, 6.9%]	6 (3.8%) [1.6, 8.6%]	
Country of origin	371				0.4
Portugal		267 (72%) [67, 76%]	159 (74%) [68, 80%]	108 (69%) [61, 76%]	
Latin America		77 (21%) [17, 25%]	43 (20%) [15, 26%]	34 (22%) [16, 29%]	
Africa		16 (4.3%) [2.6, 7.1%]	8 (3.7%) [1.7, 7.5%]	8 (5.1%) [2.4, 10%]	
Other		11 (3.0%) [1.6, 5.4%]	4 (1.9%) [0.60, 5.0%]	7 (4.5%) [2, 9.3%]	
Migrant status	371				0.2
Native		268 (72%) [67, 77%]	160 (75%) [68, 80%]	108 (69%) [61, 76%]	
Migrant		103 (28%) [23, 33%]	54 (25%) [20, 32%]	49 (31%) [24, 39%]	
District of residence	369				0.2
Lisboa		194 (53%) [47, 58%]	116 (54%) [48, 61%]	78 (50%) [42, 58%]	
Porto		58 (16%) [12, 20%]	32 (15%) [11, 21%]	26 (17%) [11, 24%]	
Faro		38 (10%) [7.5, 14%]	24 (11%) [7.5, 16%]	14 (9%) [5.2, 15%]	
Setúbal		32 (8.7%) [6.1, 12%]	21 (9.9%) [6.3, 15%]	11 (7.1%) [3.7, 13%]	
Other		47 (13%) [9.6, 17%]	20 (9.4%) [6.0, 14%]	27 (17%) [12, 24%]	
School level	367				0.8
Third level (up to 9th degree)		58 (15.8%) [12, 20%]	32 (15%) [11, 21%]	26 (17%) [12, 24%]	
Secondary (12th degree)/Technical Specialization		153 (41.7%) [37, 47%]	89 (42%) [35, 49%]	64 (42%) [34, 50%]	
Higher education (bachelor, master, PhD)		156 (42.5%) [37, 48%]	93 (43%) [37, 50%]	63 (41%) [33, 49%]	
Current occupation	359				0.2
Employed		273 (76%) [71, 80%]	164 (78%) [71, 83%]	109 (74%) [66, 80%]	
Unemployed		56 (15.6%) [12, 20%]	27 (13%) [8.7, 18%]	29 (20%) [14, 27%]	
Other		30 (8.4%) [5.8, 12%]	20 (9.5%) [6.0, 14%]	10 (6.8%) [3.5, 12%]	
Current income	337				0.2
Insufficient (≤500€)		64 (19%) [15, 24%]	43 (22%) [16, 28%]	21 (15%) [10, 23%]	
Minimum wage (501–1,000€)		173 (51%) [46, 57%]	105 (52%) [45, 60%]	68 (50%) [41, 58%]	
Average (1001–2000€)		77 (23%) [19, 28%]	42 (21%) [16, 27%]	35 (26%) [19, 34%]	
Above average (>2000€)		23 (6.8%) [4.5, 10%]	10 (5%) [2.6, 9.3%]	13 (9.5%) [5.4, 16%]	

IQR, Interquartile Range; CI, Confidence Interval.

### 3.2 Sexual behavioral, testing, prophylaxis, and STIs

As sexual partners, 87% (95% CI: 83–90%) of men reported to have sex with other men and 13% (95% CI: 9.6–17%) with both men and women. Men reported to have found sexual partners primarily on mobile apps (67, 95% CI: 62–72%) and online (64, 95% CI: 58–69%), with an overall proportion of unprotected anal sex in the last 12 months of 65% (95% CI: 60–70%). In the last unprotected sex with an occasional partner, 24% (95% CI: 18–31%) of men asked their partner about their HIV serological status. Of those who did not ask, 68% (95% CI: 56–78%) assumed the occasional partner was HIV-negative. In the last 12 months, during unprotected sex, 57% (95% CI: 50–64%) of men drank alcohol, and 46% (95% CI: 40–53%) did illicit drugs (Table 2). No significant differences regarding sexual behaviors was found between MSM with LP and Non LP.

**Table 2:** Sexual behaviors of MSM overall, non-late presenters (NonLP) and late presenters (LP).

	*N*	Overall	NonLP (*N* = 214)	LP (*N* = 157)	*p*-value
		*N* (%) [95% CI] Median (IQR)	*N* (%) [95% CI] Median (IQR)	*N* (%) [95% CI] Median (IQR)	
Sexual Behaviors
Sexual partners	361				0.12
Men		315 (87%) [83, 90%]	189 (90%) [84, 93%]	126 (84%) [77, 89%]	
Men and women		46 (13%) [9.6, 17%]	22 (10%) [6.8, 16%]	24 (16%) [11, 23%]	
Meets sexual partners in coffee shops/bars/Discos (yes)	339	189 (56%) [50, 61%]	118 (58%) [50, 64%]	71 (53%) [44, 62%]	0.4
Meets sexual partners in saunas (yes)	327	77 (24%) [19, 29%]	47 (24%) [18, 31%]	30 (23%) [16, 31%]	0.8
Meets sexual partners in cruising circuits (yes)	330	86 (26%) [21, 31%]	54 (28%) [22, 35%]	32 (24%) [17, 32%]	0.4
Meets sexual partners online (yes)	335	214 (64%) [58, 69%]	133 (66%) [59, 73%]	81 (60%) [52, 69%]	0.3
Meets sexual partners in mobile apps (yes)	335	224 (67%) [62, 72%]	135 (68%) [60, 73%]	89 (65%) [57, 73%]	0.6
Unprotected anal sex (last 12 months) (yes)	340	221 (65%) [60, 70%]	135 (67%) [60, 73%]	86 (63%) [54, 71%]	0.5
Unprotected insertive anal sex (last 12 months) (yes)	211	165 (78%) [72, 83%]	104 (79%) [71, 85%]	61 (77%) [66, 86%]	0.8
Unprotected receptive anal sex (last 12 months) (yes)	214	185 (86%) [81, 91%]	118 (87%) [80, 92%]	67 (85%) [75, 92%]	0.6
Unprotected anal or vaginal sex with a woman (last 12 months) (yes)	363	51 (14%) [11, 18%]	31 (15%) [10, 20%]	20 (13%) [8.5, 20%]	0.7
Condom use in trios/group sex (last 12 months)	363				0.5
Didn’t have sex in group		213 (59%) [53, 64%]	119 (56%) [49, 63%]	94 (62%) [54, 70%]	
Yes		90 (25%) [21, 30%]	56 (26%) [21, 33%]	34 (23%) [16, 30%]	
No		60 (17%) [13, 21%]	37 (17%) [13, 23%]	23 (5%) [10, 22%]	
Asked steady partner his serologic status for HIV (Last unprotected sex) (yes)	253	91 (36%) [30, 42%]	58 (37%) [30, 46%]	33 (34%) [25, 44%]	0.5
If yes, his status was	88				0.7
HIV_negative		51 (58%) [47, 68%]	32 (57%) [43, 70%]	19 (59%) [41, 76%]	
HIV_positive		23 (26%) [18, 37%]	15 (27%) [16, 41%]	8 (25%) [12, 44%]	
Unknown		11 (12%) [6.7, 22%]	8 (14%) [6.8, 27%]	3 (9.4%) [2.5, 26%]	
Do not_remember		3 (3.4%) [0.88, 10%]	1 (1.8%) [0.09, 11%]	2 (6.3%) [1.1, 22%]	
If not, supposed to be	120				0.5
HIV_negative		67 (56%) [46, 65%]	41 (55%) [43, 67%]	26 (57%) [41, 71%]	
HIV_positive		19 (16%) [10, 24%]	13 (18%) [10, 29%]	6 (13%) [5.4, 27%]	
Unknown		10 (8.3%) [4.3, 15%]	4 (5.4%) [1.7, 14%]	6 (13%) [5.4, 27%]	
Do not_remember		24 (20%) [13, 28%]	16 (22%) [13, 33%]	8 (17%) [8.3, 32%]	
Asked occasional partner his serologic status for HIV (Last unprotected sex) (yes)	181	43 (24%) [18, 31%]	26 (23%) [16, 32%]	17 (25%) [16, 37%]	0.8
If yes, his status was	36				> 0.9
HIV_negative		28 (78%) [60, 89%]	18 (78%) [56, 92%]	10 (77%) [46, 94%]	
HIV_positive		3 (8.3%) [2.2, 24%]	2 (8.7%) [1.5, 30%]	1 (7.7%) [0.4, 38%]	
Unknown		5 (14%) [5.2, 30%]	3 (13%) [3.4, 35%]	2 (15%) [2.7, 46%]	
If not, supposed to be	77				> 0.9
HIV_negative		52 (68%) [56, 78%]	35 (69%) [54, 80%]	17 (65%) [44, 82%]	
HIV_positive		7 (9.1%) [4.0, 18%]	5 (9.8%) [3.7, 22%]	2 (7.7%) [1.3, 27%]	
Unknown		18 (23%) [15, 35%]	11 (22%) [12, 36%]	7 (27%) [12, 48%]	
Consumed alcohol in unprotected sex (Yes)	222	127 (57%) [50, 64%]	81 (59%) [50, 67%]	46 (54%) [43, 65%]	0.5
ChemSex in unprotected sex (Yes)	222	103 (46%) [40, 53%]	67 (50%) [41, 58%]	36 (41%) [31, 52%]	0.2

IQR, Interquartile Range; CI, Confidence Interval.

Regarding screening habits for HIV infection, 45% (95% CI: 40–51%) of men reported to have been tested for HIV more than once a year, 33% (95% CI: 29–39%) tested once a year or less, and 21% (95% CI: 17–26%) never got tested before the diagnosis (Table 3). As expected the proportion of MSM who never got tested before the diagnosis was significantly higher in MSM with LP (30, 95% CI: 23–38%) than Non LP (15, 95% CI:11–21%). Conversely, the proportion of MSM who tested more than once per year with Non LP (51, 95% CI: 44–58%) was higher than in MSM with LP (38, 95% CI: 30–46%).

**Table 3:** Testing and prophylaxis in MSM overall, non-late presenters (NonLP) and late presenters (LP).

	*N*	Overall	NonLP (*N* = 214)	LP (*N* = 157)	*p*-value
		*N* (%) [95% CI] Median (IQR)	*N* (%) [95% CI] Median (IQR)	*N* (%) [95% CI] Median (IQR)	
Testing and Prophylaxis
HIV testing frequency	356				0.002
More than once per year		161 (45%) [40, 51%]	105 (51%) [44, 58%]	56 (38%) [30, 46%]	
Once per year		119 (33%) [29, 39%]	71 (34%) [28, 41%]	48 (32%) [25, 40%]	
Never got tested		76 (21%) [17, 26%]	31 (15%) [11, 21%]	45 (30%) [23, 38%]	
Pre–exposure prophylaxis (ever)	357				0.6
No		304 (85%) [81, 89%]	180 (87%) [81, 91%]	124 (83%) [76, 89%]	
Yes		15 (4.2%) [2.5, 7.0%]	7 (3.4%) [1.5, 7.1%]	8 (5.4%) [2.5, 11%]	
Do not know what it is		38 (11%) [7.7, 14%]	21 (10%) [6.5, 15%]	17 (11%) [7, 18%]	
Post–exposure prophylaxis (ever)	359				0.5
No		295 (82%) [78, 86%]	175 (84%) [78, 89%]	120 (79%) [72, 85%]	
Yes		23 (6.4%) [4.2, 9.6%]	11 (5.3%) [2.8, 9.5%]	12 (7.9%) [4.4, 14%]	
Do not know what it is		41 (11%) [8.4, 15%]	22 (11%) [6.9, 16%]	19 (13%) [7.9, 19%]	

IQR, Interquartile Range; CI, Confidence Interval.

As for STIs, 42% (95% CI: 37–47%) of men reported having had at least 1 STI in the past, with higher proportion in MSM with LP (48, 95% CI: 40–56%) than Non LP (37, 95% CI: 31–44%) (Supplementary Table S1).

### 3.3 Clinical information and viral genomics

Regarding status at presentation for care, 58% (95% CI: 52–63%) were classified as NonLP and 42.3% (95% CI: 37–48%) were classified as LP. Overall, there was 7.3% (95% CI: 4.9–11%) classified as LPAD. The CD4 median count for NonLP was 532 cells/ μL (IQR: 442–668) and 211 cells/mm3 (IQR: 100, 292) for LP. The viral load was, as expected, significantly different between Non-LP and LP status, with 50% (95% CI: 43–57%) of Non-LP with 10,000–100,000 copies/mL and 60% (95% CI: 52–68%) of LP with ≥100,000 copies/mL (Supplementary Table S2).

The most frequent subtype was B with 60% of cases (95% CI: 55–65%), followed by A1 with 12% (95% CI: 9–16%) of cases. The overall prevalence of TDR in this population was 8.2% (95% CI: 5.6–12%). Higher ambiguity rate in MSM with LP (0.61, IQR: 0.23–1.3) than Non LP (0.15, IQR: 0–0.46). No other significant differences were observed in the genomic characteristics between the two groups (Supplementary Table S3).

### 3.4 Sociodemographic, behavioral, testing, prophylaxis, and STIs factors associated to LP

**Figure 1:**
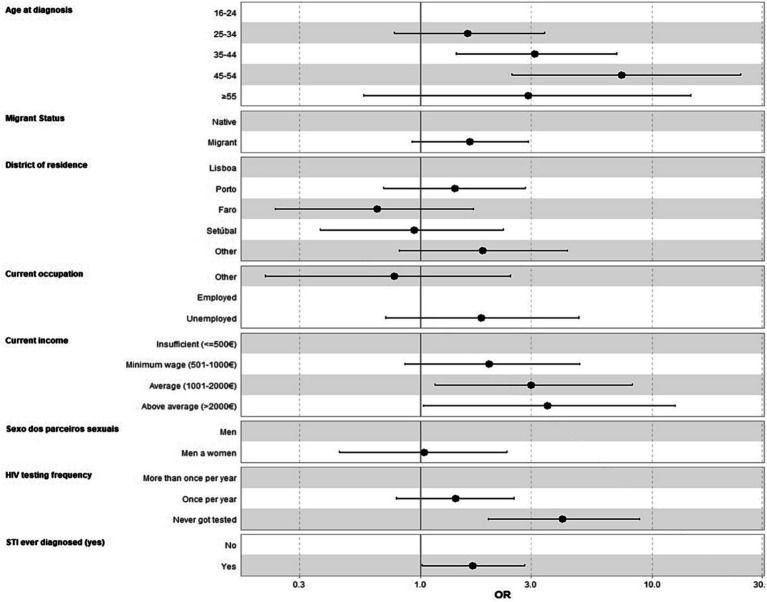
Adjusted logistic regression analysis of sociodemographic, behavior, testing, prophylaxis and STIs factors associated with HIV late presentation status (LP). STI, Sexual transmitted infections.

In the adjusted logistic regression analysis, the sociodemographic, behavioral, testing, prophylaxis and STIs factors (Table 4) significantly associated with LP status were age at diagnosis (35–44 compared to 18–24, OR = 3.11, 95% CI [1.43, 7.02]; 45–54 compared to 18–24, OR = 7.38, 95% CI [2.47, 24.2]), Current income (Average (1001–2000€) compared to Insufficient (≤500€), OR = 2.99, 95% CI [1.15, 8.19]; Above average (>2000€) compared to Insufficient (≤500€), OR = 3.53, 95% CI [1.03, 12.5]), HIV testing frequency (Never got tested compared to More than once per year, OR = 4.08, 95% CI [1.96, 8.78]), and STI ever diagnosed (Yes compared to No, OR = 1.68, 95% CI [1.01, 2.81]). Migrant status, District of residence, Current occupation and Sexual partners were included in the multivariate model but were not significantly associated with LP status (Figure 1).

**Table 4:** Univariate and multivariate logistic regression analysis of sociodemographic, behavior, testing, prophylaxis, and STIs factors associated with HIV late presentation (LP).

Univariate and multivariate logistic regression analysis of sociodemographic behavior testing prophylaxis and STIs factors associated with HIV late presenter (LP) status				
	Univariate		Multivariate (*N* = 312)	
	OR (95% CI)	*p-*value	OR (95% CI)	*p-*value
Sociodemographics
Diagnosis Year
2014–2016	1			
2017–2019	1.26 (0.82–1.95)	0.29		
Age at diagnosis	1.05 (1.02–1.07)	0.001		
Age at diagnosis (groups)
18–24	1		1	
25–34	1.18 (0.66–2.14)	0.58	1.59 (0.77–3.43)	0.2
35–44	2.69 (1.44–5.13)	0.002	3.11 (1.43–7.02)	0.005
45–54	4.91 (2.12–12)	<0.001	7.38 (2.47–24.2)	< 0.001
≥55	1.93 (0.57–6.42)	0.28	2.91 (0.57–14.7)	0.2
Country of origin
Portugal	1			
Latin America	1.16 (0.69–1.94)	0.56		
Africa	1.47 (0.53–4.12)	0.45		
Other	2.58 (0.76–10)	0.14		
Migrant status
Native	1			
Migrant	1.34 (0.85–2.12)	0.2	1.63 (0.92–2.91)	0.1
District of residence
Lisboa	1		1	
Porto	1.21 (0.67–2.18)	0.53	1.4 (0.69–2.83)	0.3
Faro	0.87 (0.41–1.76)	0.7	0.65 (0.23–1.69)	0.4
Setúbal	0.78 (0.34–1.68)	0.53	0.93 (0.37–2.27)	0.9
Other	2.01 (1.06–3.87)	0.034	1.86 (0.81–4.29)	0.15
School level
Third level (up to 9th degree)	1			
Secondary (12th degree)/Technical Specialization	0.89 (0.48–1.63)	0.69		
Higher education (bachelor, master, PhD)	0.83 (0.45–1.54)	0.56		
Current occupation
Employed	1		1	
Unemployed	1.62 (0.91–2.89)	0.1	1.82 (0.71–4.81)	0.2
Other	0.75 (0.33–1.63)	0.48	0.77 (0.21–2.44)	0.7
Current income
Insufficient (≤500€)	1		1	
Minimum wage (501–1,000€)	1.33 (0.73–2.46)	0.36	1.98 (0.85–4.84)	0.12
Average (1001–2000€)	1.71 (0.86–3.43)	0.13	2.99 (1.15–8.19)	0.028
Above average (>2000€)	2.66 (1.01–7.22)	0.049	3.53 (1.03–12.5)	0.047
Behaviors
Sexual partners				
Men	1		1	
Men and women	1.64 (0.88–3.06)	0.12	1.03 (0.45–2.35)	>0.9
Meets sexual partners in coffee shops/bars/Discos
No	1			
Yes	0.83 (0.54–1.29)	0.41		
Meets sexual partners in saunas
No	1			
Yes	0.93 (0.55–1.56)	0.77		
Meets sexual partners in cruising circuits
No	1			
Yes	0.81 (0.49–1.34)	0.42		
Meets sexual partners online
No	1			
Yes	0.78 (0.5–1.23)	0.29		
Meets sexual partners in mobile apps
No	1			
Yes	0.9 (0.57–1.43)	0.65		
Unprotected anal sex (last 12 months)
No	1			
Yes	0.85 (0.54–1.34)	0.48		
Unprotected insertive anal sex (last 12 months)
No	1			
Yes	0.91 (0.47–1.81)	0.79		
Unprotected receptive anal sex (last 12 months)
No	1			
Yes	0.8 (0.36–1.82)	0.59		
Unprotected anal or vaginal sex with a woman (last 12 months)
No	1			
Yes	0.89 (0.48–1.62)	0.71		
Condom use in trios/group sex (last 12 months)
Didn’t have sex in group	1			
Yes	0.77 (0.46–1.27)	0.31		
No	0.79 (0.43–1.41)	0.42		
Asked steady partner his serologic status for HIV (Last unprotected sex)
No	1			
Yes	0.85 (0.5–1.44)	0.55		
Asked occasional partner his serologic status for HIV (Last unprotected sex)
No	1			
Yes	1.12 (0.55–2.24)	0.76		
Consumed alcohol in unprotected sex				
No	1			
Yes	0.82 (0.47–1.41)	0.46		
ChemSex in unprotected sex
No	1			
Yes	0.72 (0.41–1.23)	0.23		
Testing and Prophylaxis
HIV testing frequency
More than once per year	1		1	
Once per year	1.27 (0.78–2.07)	0.34	1.41 (0.79–2.53)	0.2
Never got tested	2.72 (1.56–4.8)	<0.001	4.08 (1.96–8.78)	<0.001
Pre–exposure prophylaxis (ever)
No	1			
Yes	1.66 (0.58–4.84)	0.34		
Do not know what it is	1.18 (0.59–2.31)	0.64		
Post–exposure prophylaxis (ever)
No	1			
Yes	1.59 (0.68–3.78)	0.28		
Do not know what it is	1.26 (0.65–2.43)	0.49		
STIs
STI ever diagnosed
No	1		1	
Yes	1.57 (1.03–2.39)	0.036	1.68 (1.01–2.81)	0.046

### 3.5 Clinical and viral genomics factors associated to LP

**Figure 2:**
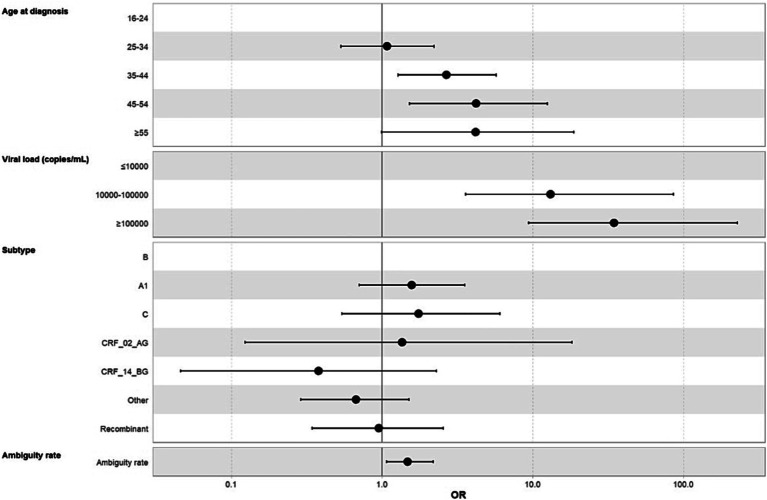
Adjusted logistic regression analysis of clinical and genomic factors associated with HIV late presentation status (LP). STI, Sexual transmitted infections.

In a second logistic regression model to study the association between clinical and viral genomic factors and LP status (Table 5), viral load (10,000–100,000 copies/mL compared to ≤10,000, OR = 12.9, 95% CI [3.54, 84.2]; ≥100,000 compared to ≤10,000, OR = 33.6, 95% CI [9.14, 220]) and the ambiguity rate (1.52, IQR: 1.09–2.24) were significantly associated with LP status. The existence of TDR and HIV subtype were not significantly associated with LP status. Age at diagnosis was included in the adjusted model and was significantly associated with LP status (35–44 compared to 18–24, OR = 2.67, 95% CI [1.28, 5.7]; 45–54 compared to 18–24; OR = 4.11, 95% CI [1.49, 12.1]) (Figure 2).

**Table 5:** Univariate and multivariate logistic regression analysis of clinical and genomic factors associated with HIV late presentation (LP).

Univariate and multivariate logistic regression analysis of clinical and genomic factors associated with HIV late presenter (LP) status				
	Unadjusted		Adjusted (*N* = 325)	
	OR (95% CI)	*p-*value	OR (95% CI)	*p-*value
Clinical
Age at diagnosis (groups)
18–24	1		1	
25–34	1.18 (0.66–2.14)	0.58	1.09 (0.54–2.23)	0.8
35–44	2.69 (1.44–5.13)	0.002	2.67 (1.28–5.7)	0.01
45–54	4.91 (2.12–12)	< 0.001	4.11 (1.49–12.1)	0.008
≥55	1.93 (0.57–6.42)	0.28	4.18 (1–18.6)	0.053
Viral load (copies/mL)
≤10,000	1		1	
10,000–100,000	3.06 (1.41–7.39)	0.007	12.9 (3.54–84.2)	< 0.001
≥100,000	9.62 (4.46–23.3)	< 0.001	33.6 (9.14–220)	< 0.001
HLA-B57
Negative	1			
Positive	0.36 (0.05–1.47)	0.2		
Genomic
Any SDRM
No	1			
Yes	1.29 (0.58–2.85)	0.53		
Subtype
B	1		1	
A1	1.15 (0.57–2.27)	0.69	1.57 (0.7–3.51)	0.3
C	2.20 (0.83–6.22)	0.12	1.76 (0.54–6.05)	0.4
Other	0.84 (0.43–1.6)	0.60	0.66 (0.31–1.37)	0.3
Recombinant	0.93 (0.39–2.16)	0.88	0.95 (0.35–2.55)	>0.9
B vs. non-B
B	1			
Non-B	1.08 (0.69–1.69)	0.73		
Ambiguity rate	1.73 (1.25–2.5)	0.002	1.52 (1.09–2.24)	0.024
Recentness
Chronic	1			
Recent	0.26 (0.17–0.42)	< 0.001		

STI, Sexual transmitted infections.

## 4 Discussion

In this study we identified sociodemographic, behavioral, clinical, and viral genomic characteristics of the MSM population diagnosed with HIV in Portugal between 2014 and 2019, and analyzed determinants of LP.

Overall, we have found that LP status was associated with specific sociodemographics, behavioral and clinical factors when compared to NonLP. LP was associated with increased age at diagnosis, higher current income, lower HIV testing frequency, previous diagnosis of STIs, increased viral load and higher ambiguity rate.

Clinically, this study found that 42.3% of patients were LP, of which 17.2% were LPAD. These proportions are lower than the ones on the Portuguese 2020–2021 HIV/AIDS Report (27), which registered 50.9% of MSM with LP, of which 28.8% of LPAD. We can hypothesize that this difference is due to the fact that 53% of MSM in our sample resided in the Lisbon metropolitan area and 16% in the Oporto metropolitan area, the two largest cities in Portugal, where there are a greater number of HIV screening centers (28) that are better geared toward this population with anonymous and community/peer services (29). Because screening for MSM is more readily available in these cities, this can have an impact on the proportion of late presenters. Another possible reason is that HIV screening, diagnosis and presentation for care may have been delayed in 2020–2021 as a result of the COVID-19 pandemic’s effects on healthcare access and use. The management of COVID-19 patients overburdened healthcare systems and practitioners, which may have limited capacity for other health services, including HIV related care. Access to HIV testing and prevention clinics may also have been more challenging due to social isolation policies and gathering-related limitations (30, 31). Comparing our findings to other European studies, we find that this LP proportion in MSM is higher to that reported in other countries, such as Spain (36.4%) (32), England (30%) (9), and overall for MSM LP in Europe (38.4%) (8). This suggests that there is still a significant amount of work to be done in terms of evaluating and providing screening services for this community.

In Portugal, access to HIV screening is widespread and often anonymous and confidential. It is available in a variety of channels (medical prescription, voluntarily at point-of-care settings, self-testing) and contexts (primary healthcare, hospital healthcare, Non-Governmental Organizations, and community pharmacies, among others) to reach the most significant number of people and prevent missing out on opportunities for diagnosis and link to care (27). We found that most MSMs in this study reported to have tested for HIV twice annually or more (45% (95% CI: 40–51%)) before their HIV diagnosis. This can be explained by social network-based strategies and community-based testing settings that can increase HIV testing and status awareness among MSM (33). Nevertheless, 21% (95% CI: 17–26%) of MSM had never been tested before the diagnosis. This proportion aligns with findings from other studies in Europe, including EMIS 2017 (21%) (34), the Netherlands (19.3%) (35) and Norway (20.1%) (36). Complex, intertwined psychosocial barriers affect HIV testing in MSM, such as anticipated perception of stigma after an HIV diagnosis, fear of judgment from partners and family or testing providers, low-risk perception, beliefs about HIV treatments, and avoidance of psychologically threatening information (37–40). Reinforcing the validity of our study design and questionnaire’s reliability, we found an expected association of MSM who had never tested before the diagnosis with LP (OR = 4.08, 95% CI [1.96, 8.78]). People who have never been tested for HIV are less likely to be aware of their HIV status, with a higher risk of developing AIDS and other HIV-related complications and in an increased risk of transmitting the virus to others (29, 41). Further studies should explore the factors associated with lack of HIV testing among MSM, regardless of the wide availability of HIV screening in Portugal. There is a need for additional strategies to increase HIV testing among those who have never been tested and are being left out of the multiple approaches for testing available for MSM in Portugal.

Migrants, a population that classically has lower access to health care services, represent 28% of our sample. Although the problem of never having been tested before concerns both natives and migrants in our data, and no association was found between migrant status and LP status, the migrants group faces a specific set of barriers regarding HIV screening, such as a lack of HIV-prevention knowledge, language barriers, uncertainty about their rights to healthcare and where to go for testing, access to care, fears regarding the resident status and structural constraints for MSM migrants (9, 42, 43). This might suggest that screening services in Portugal are effectively reaching this vulnerable population. Nevertheless, this lack of association between LP and migrant status contradicts previous research that showed African migrants were more likely to be late presenters owing to limited access to healthcare, poverty, and stigma (15, 44–46). One possible explanation is that other studies included African women and heterosexual male migrants. Our sample contained only 16 (4%) African MSM, which can be explained by the fact that MSM from Africa may not disclose they have had sex with men to healthcare practitioners (47, 48) and were not included in our sample. This lack of association in our study is also likely to be due to this low number of participants from Africa in our sample.

The median age of newly infected MSM’s in our study aligns with previous reports. The WHO HIV/AIDS surveillance in Europe estimates that, in 2021, the age at diagnosis was under 39 years old in more than 60% of MSM (1). The Portuguese 2020–2021 HIV/AIDS Report registered a similar median age of 31 years old (IQR 26–38) (27). The fact that younger MSM are more likely to engage in behaviors that increase their risk of HIV transmission, such as having multiple sexual partners, engaging in unprotected sex and drug use (49, 50), can explain this age pattern. On the other hand, we have found an association between older MSM and LP, reinforcing previous studies. The association between older MSM and LP has been repeatedly demonstrated in research (15, 51–54). There are some explanations for why older MSM may be at increased risk of LP. Studies have shown that older MSM may be less likely to get tested for HIV. This may be because of patient related factors or health care related factors. As for patient related factors, they do not perceive themselves to be at risk (55, 56), they may misinterpret HIV symptoms as age-related (56), they may feel excluded from HIV testing campaigns (56) and older MSM may have little or no connection to the gay community (57). On the other hand, on the health care side, primary care settings are less likely to offer HIV testing to older MSM (58) and health workers may also misinterpret symptoms assuming those as age-related and pertain from performing an HIV test (58).

An important finding in our study is that MSM with higher income were associated with a higher probability of LP (Average (1001–2000€), OR = 2.99, 95% CI [1.15, 8.18]; Above average (>2000€), OR = 3.53, 95% CI [1.03, 12.5]). The adjusted analysis reinforces the univariate results for this association which were also significant (please refer to Table 4), emphasizing the strength of this finding. Existing research does not describe whether economic status is associated with LP. To our knowledge, no study with MSM included the monthly income of each participant as a variable in its analysis. There are studies with scores that indicate the participant’s socioeconomic status, constructed based on the average income of households of the region where the participant lives and other measures such as educational level, employment status, and other factors. One study in the Netherlands included a socioeconomic score that considered the average income per household in a given postal code area, the percentage of households with low income, without paid jobs, and with low education level, and found no association with LP (59). Another study in Germany included the German Index of Socioeconomic Deprivation of their residential area, based on the subdimensions of education, occupation, and income. The study concluded that only the MSM who live in the countryside versus urban areas were affected by socioeconomic deprivation, and there was no impact on MSM from towns or major cities (60). The association between higher income and LP can be explained by stigma surrounding HIV. We can hypothesize that there may be differences in HIV-related stigma and discrimination across income levels. People with higher income may experience different forms of stigma and discrimination socially and professionally related to an HIV diagnosis, which can also contribute to delays in testing. The reasons why people with higher monthly income are at a higher risk of LP may be complex and multifactorial. For example, this finding could also be a surrogate of older age, as older MSM should have higher income. But the multicollinearity assessment of the adjusted regression model in our analysis indicated otherwise, showing no multicollinearity between variables (refer to Additional File 1). Future studies should address this with a comprehensive approach considering social, economic, and professional factors.

There are some limitations associated to this study to acknowledge. Firstly, the new definition of LP (61) was not applied because information on the last negative test was not consistently available for most participants. LP was based on the consensus definition (10) and, as such, may be overestimated due to a transient decrease of the CD4 count upon seroconversion period and in the early stage of infection. To overcome that limitation, we used ambiguity levels of genomic sequences to further define recentness of infection. Secondly, 69% of the MSM in this sample resided in the two largest cities in Portugal and MSM sexual behaviors and HIV testing habits may differ from non-urban areas (62–64).

Our study reinforces the previously established connections between late presentation for HIV care and factors such as increased age and low testing frequency. However, we also highlight a previously overlooked group of men who have sex with men (MSM) with higher incomes, who are also being left out in the efforts to increase HIV screening and to achieve the 95-95-95 targets toward the 2030 Agenda for Sustainable Development (41).

## 5 Conclusion

Specific subgroups of the MSM population, such older MSM, with higher income and lower HIV testing frequency, are not being targeted by community and clinical screening services. Overall, targeted public health measures should be strengthened toward these subgroups, through strengthened primary care testing, expanded access to PrEP, information and promotion of HIV self-testing and more inclusive and accessible health services.

## Data availability statement

The original contributions presented in the study are included in the article/Supplementary materials, further inquiries can be directed to the corresponding author.

## Ethics statement

The studies involving humans were approved by the Ethics Committees of all participating hospitals. The studies were conducted in accordance with the local legislation and institutional requirements. The participants provided their written informed consent to participate in this study.

## Author contributions

RA: Conceptualization, Data curation, Formal analysis, Validation, Writing – original draft, Writing – review & editing. VP: Data curation, Investigation, Methodology, Validation, Writing – review & editing. MNSM: Data curation, Validation, Writing – review & editing. AS: Resources, Writing – review & editing. AD: Resources, Writing – review & editing. BA: Resources, Writing – review & editing. CP: Resources, Writing – review & editing. CK: Resources, Writing – review & editing. CR: Resources, Writing – review & editing. CC: Resources, Writing – review & editing. CM: Resources, Writing – review & editing. DF: Resources, Writing – review & editing. EG: Resources, Writing – review & editing. ET: Resources, Writing – review & editing. FáM: Resources, Writing – review & editing. FaR: Resources, Writing – review & editing. FeM: Resources, Writing – review & editing. FeR: Resources, Writing – review & editing. GG: Resources, Writing – review & editing. HR: Resources, Writing – review & editing. IC: Resources, Writing – review & editing. IG: Resources, Writing – review & editing. JSi: Resources, Writing – review & editing. JO: Resources, Writing – review & editing. JF: Resources, Writing – review & editing. JP: Resources, Writing – review & editing. JSa: Resources, Writing – review & editing. JSo: Resources, Writing – review & editing. SF: Resources, Writing – review & editing. KM: Resources, Writing – review & editing. LP: Resources, Writing – review & editing. MA: Resources, Writing – review & editing. MG: Resources, Writing – review & editing. MJM: Resources, Writing – review & editing. MM: Resources, Writing – review & editing. MS: Resources, Writing – review & editing. MC: Resources, Writing – review & editing. NM: Resources, Writing – review & editing. OC: Resources, Writing – review & editing. PPa: Resources, Writing – review & editing. PPr: Resources, Writing – review & editing. PR: Resources, Writing – review & editing. RP: Resources, Writing – review & editing. RT: Resources, Writing – review & editing. RC: Resources, Writing – review & editing. RC-R: Resources, Writing – review & editing. RoS: Resources, Writing – review & editing. RuS: Resources, Writing – review & editing. SN: Resources, Writing – review & editing. TF: Resources, Writing – review & editing. TB: Resources, Writing – review & editing. DS: Resources, Writing – review & editing. LM: Resources, Writing – review & editing. MO: Investigation, Methodology, Writing – review & editing. PG: Investigation, Resources, Writing – review & editing. MP: Data curation, Formal analysis, Investigation, Methodology, Validation, Writing – review & editing. AA: Conceptualization, Formal analysis, Funding acquisition, Methodology, Project administration, Supervision, Writing – original draft, Writing – review & editing.

## Group members of BESTHOPE

Ana Bandeiras, Ana Pimenta, Anabela Granado, André Gomes, António Maio, Catarina Messias, Celina Bredes, Daniel Simões, Diana Seixas, Diva Trigo, Edite Mateus, Fátima Gonçalves, Filipa Azevedo, Francisco Vale, Henriqueta Pereira, Inês Siva, Isabel Casella, Isabel Diogo, Isabel Neves, Joana Sá, Joana Simões, Joana Granado, Joana Vasconcelos, João Cabo, João Pereira-Vaz, João Domingos, João Torres, Joaquim Cabanas, Johana Jesus, José Melo Cristino, Karen Pereira, Luís Caldeira, Luís Mendão, Luísa Sêco, Lurdes Correia, Manuela Simão, Maria Saudade Ivo, Mariana Pessanha, Marta Feijó, Margarida Cardoso, Nildelema Malaba, Nádia Gomes, Natália Patrício, Nuno Luís, Nuno Janeiro, Patrícia Carvalho, Paula Brito, Pedro Simões, Rosário Prazos, Sara Lino, Sara Casanova, Sofia Pinheiro, Sónia Marques, Sofia Jordão, Sueila Martins, Telma Azevedo, Teresa Meira, Vanda Mota, and Vanda Silva.

## Footnotes

^1^ http://hivdb.stanford.edu

## In memoriam

We would like to thank Dr. Ricardo Camacho, who passed away in 2018, who designed and triggered this study. Without his deep involvement, this study would not have been possible.
