# Supplementary material for: Determinants of HIV late presentation among men who have sex with men in Portugal (2014–2019): who’s being left behind?
Source: Front Public Health. 2024 Feb 29;12:1336845. doi: 10.3389/fpubh.2024.1336845 (PMC10947991; doi:10.3389/fpubh.2024.1336845)
Supplement: Supplementary file 1 [file Data_Sheet_1.docx]

**Supplementary Material**

|  |  | **Overall** | **NonLP (N = 214)** | **LP (N = 157)** | **p-value** |
| --- | --- | --- | --- | --- | --- |
|  | N | N (%) [95% CI] | N (%) [95% CI] | N (%) [95% CI] |  |
|  |  | median (IQR) | median (IQR) | median (IQR) |  |
| **STIs** | | | | | |
| **STI ever diagnosed (yes)** | 367 | 153 (42%) [37%, 47%] | 79 (37%) [31%, 44%] | 74 (48%) [40%, 56%] | **0.036** |
| **Lifetime Total IST** | 153 | 1 (1, 1) | 1 (1, 1) | 1 (1, 1) | 0.7 |

**Table S1 – Sexual transmitted infections in MSM overall, non-late presenters (NonLP) and late presenters (LP). IQR – Interquartile Range; CI – Confidence Interval.**

|  |  | **Overall** | **NonLP (N = 214)** | **LP (N = 157)** | **p-value** |
| --- | --- | --- | --- | --- | --- |
|  | **N** | N (%) [95% CI] | N (%) [95% CI] | N (%) [95% CI] |  |
|  |  | median (IQR) | median (IQR) | median (IQR) |  |
| **Clinical** | | | | | |
| **CD4 count (cells/mm^3^)** | 371 | 404 (255, 575) | 532 (442, 668) | 211 (100, 292) | **<0.001** |
| **CD8 count (cells/mm^3^)** | 322 | 904 (627, 1234) | 1026 (744, 1322) | 763 (468, 1,062) | **<0.001** |
| **Viral load (copies/mL)** | 367 |  | | | **<0.001** |
| ≤10000 |  | 56 (15%) [12%, 19%] | 48 (23%) [17%, 29%] | 8 (5.2%) [2.4%, 10%] |  |
| 10000-100000 |  | 160 (44%) [38%, 49%] | 106 (50%) [43%, 57%] | 54 (35%) [27%, 43%] |  |
| ≥100000 |  | 151 (41%) [36%, 46%] | 58 (27%) [22%, 34%] | 93 (60%) [52%, 68%] |  |
| **Infection stage** | 362 |  | | | **<0.001** |
| A |  | 297 (82%) [78%, 86%] | 197 (95%) [91%, 98%] | 100 (65%) [56%, 72%] |  |
| B |  | 33 (9.1%) [6.4%, 13%] | 10 (4.8%) [2.5%, 9.0%] | 23 (15%) [9.8%, 22%] |  |
| C |  | 32 (8.8%) [6.2%, 12%] |  | 32 (21%) [15%, 28%] |  |
| **HLA-B57** | 308 |  | | | 0.3 |
| Negative |  | 298 (97%) [94%, 98%] | 176 (96%) [91%, 98%] | 122 (98%) [94%, 100%] |  |
| Positive |  | 10 (3.2%) [1.7%, 6.1%] | 8 (4.3%) [2.0%, 8.7%] | 2 (1.6%) [0.28%, 6.3%] |  |

**Table S2 – Clinical characteristics of MSM overall, non-late presenters (NonLP) and late presenters(LP). IQR – Interquartile Range; CI – Confidence Interval.**

|  |  | **Overall** | **NonLP (N = 214)** | **LP (N = 157)** | **p-value** |
| --- | --- | --- | --- | --- | --- |
|  | **N** | N (%) [95% CI] | N (%) [95% CI] | N (%) [95% CI] |  |
|  |  | median (IQR) | median (IQR) | median (IQR) |  |
| **Genomics** | | | | | |
| **Any SDRM** | 328 | 27 (8.2%) [5.6%, 12%] | 14 (7.4%) [4.3%, 12%] | 13 (9.4%) [5.3%, 16%] | 0.5 |
| **Subtype** | 328 |  | | | 0.5 |
| B |  | 197 (60%) [55%, 65%] | 115 (61%) [53%, 68%] | 82 (59%) [50%, 67%] |  |
| A1 |  | 40 (12%) [9.0%, 16%] | 22 (12%) [7.6%, 17%] | 18 (13%) [8.1%, 20%] |  |
| C |  | 18 (5.5%) [3.4%, 8.7%] | 7 (3.7%) [1.6%, 7.8%] | 11 (7.9%) [4.2%, 14%] |  |
| Other |  | 48 (15%) [11%, 19%] | 30 (16%) [11%, 22%] | 18 (13%) [8.1%, 20%] |  |
| Recombinant |  | 25 (7.6%) [5.1%, 11%] | 15 (7.9%) [4.7%, 13%] | 10 (7.2%) [3.7%, 13%] |  |
| **B vs non-B** | 328 |  | | | 0.7 |
| B |  | 197 (60%) [55%, 65%] | 115 (61%) [53%, 68%] | 82 (59%) [50%, 67%] |  |
| Non-B |  | 131 (40%) [35%, 45%] | 74 (39%) [32%, 47%] | 57 (41%) [33%, 50%] |  |
| **Ambiguity rate** | 328 | 0.31 (0.08, 0.77) | 0.15 (0, 0.46) | 0.61 (0.23, 1.30) | **< 0.001** |
| **Recentness** | 328 |  | | | **< 0.001** |
| Chronic |  | 132 (40%) [35%, 46%] | 51 (27%) [21%, 34%] | 81 (58%) [50%, 66%] |  |
| Recent |  | 196 (60%) [54%, 65%] | 138 (73%) [66%, 79%] | 58 (42%) [34%, 50%] |  |

**Table S3 – Genomic characteristics of MSM overall, non-late presenters (NonLP) and late presenters (LP). IQR – Interquartile Range; CI – Confidence Interval; SDRM – Surveillance drug resistance mutation; PI - Protease inhibitors; NRTI - Nucleoside reverse transcriptase inhibitors; NNRTI – Non-Nucleoside reverse transcriptase inhibitors.**

**Variance inflation factor (VIF)**

Multivariate logistic regression analysis of sociodemographic behavior testing prophylaxis and STIs factors associated with HIV late presenter (LP) status

GVIF Df GVIF^(1/(2*Df))

IdadeDiagnCateg 1.527710 4 1.054399

PaisDeOrigem 1.102071 1 1.049796

DistrictResidenceReduzRegLog 1.418274 4 1.044648

Current_occupation_0a3RegLog 1.716453 2 1.144611

Rendimento_0a4RegLog 1.740875 3 1.096801

People_have_sex 1.135596 1 1.065644

How_often_tested_for_HIV 1.355172 2 1.078943

Tabela1.ISTs 1.040708 1 1.020151

Multivariate logistic regression analysis of clinical and viral genomics factors associated with HIV late presenter (LP) status

GVIF Df GVIF^(1/(2*Df))

IdadeDiagnCateg 1.137228 4 1.016204

ViralLoad_Categ 1.073106 2 1.017796

SubtypeRegLogPresentersv3 1.110167 4 1.013149

Tabela2.Taxa_de_ambiguidade 1.023699 1 1.011780
